# Unusual Presentation of Metastatic Testicular Mixed-Germ Cell Tumor with Intracardiac Extension: A Case Report

**DOI:** 10.3390/jcm14103564

**Published:** 2025-05-20

**Authors:** Marlon Rojas-Cadena, Felipe Rodríguez-Arcentales, Williams Lata, Karla Mera Sacoto, Luis Guerrero, Katherin Narváez Inca, Marlon Arias-Intriago, Esteban Ortiz-Prado, Juan S. Izquierdo-Condoy

**Affiliations:** 1 Department of Cardiology, Universidad Católica del Ecuador, Quito 170525, Ecuador; 2 One Health Research Group, Universidad de las Américas, Quito 170124, Ecuador; 3 Department of Cardiology, Hospital de Especialidades Eugenio Espejo, Quito 170702, Ecuador

**Keywords:** testicular neoplasms, germ cell and embryonal neoplasms, neoplasm metastasis, heart neoplasms

## Abstract

**Background:** Testicular germ cell tumors (GCTs) are highly curable malignancies, particularly when diagnosed early. However, cardiac metastases are exceedingly rare—occurring in less than 1% of cases—and pose significant diagnostic and therapeutic challenges. Intracardiac involvement is exceptionally uncommon and typically necessitates a multidisciplinary approach for optimal management. **Objective:** To present a rare case of metastatic testicular GCT with intracardiac extension in a young male, underscoring the diagnostic complexity and therapeutic considerations of this unusual clinical scenario. **Case Report:** A 23-year-old male presented with diffuse abdominal pain, dyspnea, and a palpable right testicular mass. Imaging revealed a testicular tumor with metastases to the lungs, liver, retroperitoneal lymph nodes, and a large intracardiac mass extending from the inferior vena cava into the right atrium. Histopathology confirmed a mixed-germ cell tumor consisting of 75% seminoma, 20% embryonal carcinoma, and 5% teratoma. The patient underwent radical right orchiectomy followed by chemotherapy with the BEP regimen (bleomycin, etoposide, cisplatin). Cardiac magnetic resonance imaging confirmed the intracardiac mass, which significantly decreased in size after treatment. Serum tumor markers (AFP and β-hCG) also showed substantial post-treatment declines, corresponding with clinical improvement. **Conclusions:** This case highlights a rare presentation of metastatic testicular GCT with intracardiac involvement, emphasizing the importance of recognizing atypical metastases. Despite its complexity, the patient responded well to chemotherapy, reinforcing the effectiveness of current treatments. Long-term follow-up and a multidisciplinary approach are essential for monitoring recurrence and complications, contributing to the understanding of rare metastatic patterns and the need for further research.

## 1. Introduction

Testicular cancer is a rare malignancy, accounting for approximately 1% of all male tumors and primarily affecting younger men between the ages of 15 and 40. In 2020, nearly 74,500 cases were reported globally, reflecting a rising global incidence. Testicular germ cell tumors (TGCTs) are the most common form of testicular cancer and are classified into two main subtypes: seminomas, which constitute approximately 55–60% of cases; and non-seminomas, accounting for 40–45% [1].

While TGCTs are highly curable when diagnosed early, they have the potential to metastasize to retroperitoneal lymph nodes, brain, neck, pulmonary arteries, lungs, liver, stomach, cartilage, inferior vena cava, aorta, and heart [2]. Among these, cardiac metastases are particularly rare and present significant diagnostic and therapeutic challenges. Endocardial and intracavitary metastases are uncommon, accounting for only 3–5% of cardiac metastases detected at autopsy. Most cardiac tumors remain asymptomatic and are often discovered incidentally during imaging or postmortem examination [3].

When symptomatic, cardiac metastases typically manifest in one of three ways. Some patients present with nonspecific symptoms such as fever, unexplained weight loss, and persistent fatigue. Others develop cardiac-related symptoms, including hemodynamic compromise due to mass effect, arrhythmias, pericardial effusion, dyspnea, chest pain, or syncope. Additionally, embolic phenomena may occur, leading to thromboembolic complications in the pulmonary or systemic circulation, further complicating the clinical scenario [4,5].

The identification of intracardiac masses relies on multimodal, noninvasive imaging techniques. Advances in cardiac imaging have significantly improved both diagnostic accuracy and prognostic assessments. Transthoracic echocardiography (TTE) is often the initial imaging modality due to its widespread availability and high sensitivity in detecting small, mobile masses. Cardiac computed tomography (CT) is preferred for evaluating suspected cardiac metastases (CMEs), offering high spatial resolution for detailed anatomical assessment. Cardiac magnetic resonance (CMR) imaging is considered the most comprehensive imaging tool due to its superior soft tissue contrast, allowing for precise tumor localization, enhanced visualization of extracardiac structures, and differentiation between benign and malignant lesions. CMR is also effective in distinguishing true masses from pseudomasses and provides functional data valuable for surgical planning [4].

We present a rare case of a young man diagnosed with an intra-atrial cardiac metastasis from a testicular seminoma, accompanied by a neoplastic thrombus extending into the inferior vena cava.

## 2. Case Presentation

A 23-year-old male with a history of deep vein thrombosis (DVT) involving the inferior vena cava (IVC) and bilateral iliac veins—managed with long-term anticoagulation—presented to the emergency department with diffuse abdominal pain and progressive right testicular mass. He had no significant family history.

On physical examination, a firm, tender mass was palpated in the right hemiscrotum, without erythema or signs of trauma. The patient was hemodynamically stable but reported exertional dyspnea consistent with New York Heart Association (NYHA) Functional Class II and intermittent febrile episodes. He denied pleuritic chest pain. Pulmonary auscultation revealed clear breath sounds bilaterally, while palpation of the left chest wall elicited localized tenderness. Abdominal examination showed diffuse tenderness without peritoneal signs. Ultrasonography confirmed a right testicular mass measuring 4 × 3 cm.

**Figure 1:**
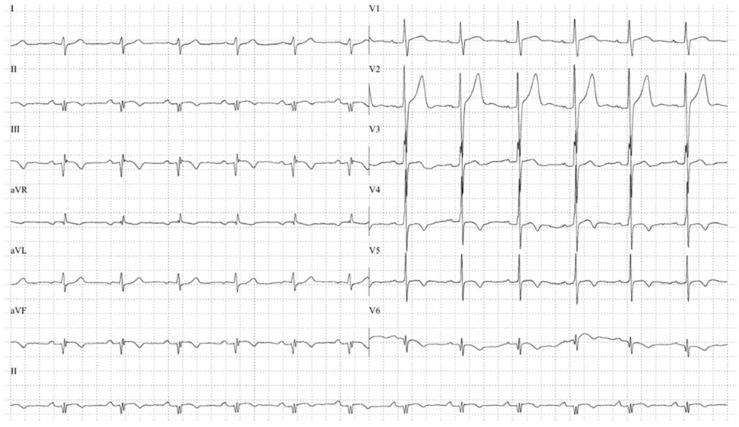
ECG reflects Q in inferior wall and in V6, and QRS fragmentation in inferior wall, V6, and aVR.

An electrocardiogram (ECG) performed at admission revealed notable abnormalities, including Q waves and QRS fragmentation in the inferior leads, suggesting electrically inactive myocardial tissue (Figure 1). Further imaging with testicular ultrasound showed an enlarged right testis with intratesticular calcifications and a heterogeneous, hypervascular mass measuring 1.7 × 0.9 cm^2^. Based on these findings, a presumptive diagnosis of testicular tumor was made, prompting the evaluation of serum tumor markers, which revealed significantly elevated alpha-fetoprotein (AFP), lactate dehydrogenase (LDH), and beta-human chorionic gonadotropin (β-hCG) levels (Table 1). Given the suspicion of malignancy, the testicular mass was successfully excised without perioperative complications.

**Figure 2:**
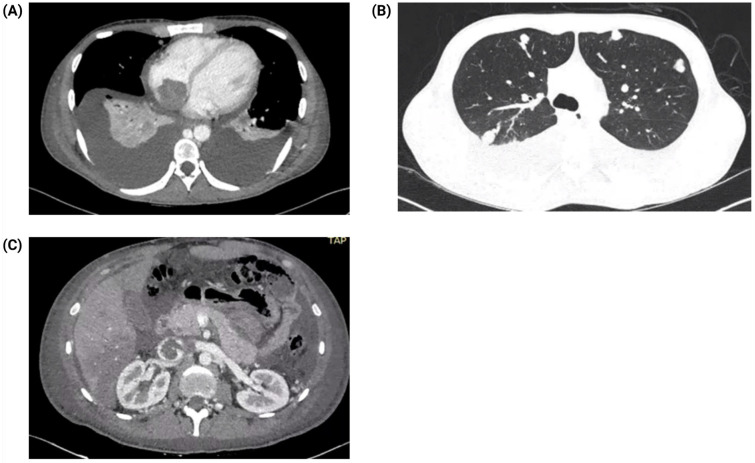
Computed tomography (CT) of the thorax and abdomen. (**A**) Bilateral pleural effusion with a large, irregular mass evident in the right atrium, suggestive of intracardiac metastasis. (**B**) Axial CT images demonstrating multiple large bilateral pulmonary nodules, consistent with extensive metastatic disease. (**C**) Contrast-enhanced CT scan showing a mass within the inferior vena cava, occupying approximately 75% of the lumen, indicating significant vascular involvement.

Postoperative computed tomography (CT) of the chest and abdomen revealed extensive metastases to both lungs, retroperitoneal lymph nodes, and liver. An irregular mass was also identified extending from the inferior vena cava into the right atrium (Figure 2). The presence of cardiac involvement raised concerns about potential hemodynamic complications.

**Figure 3:**
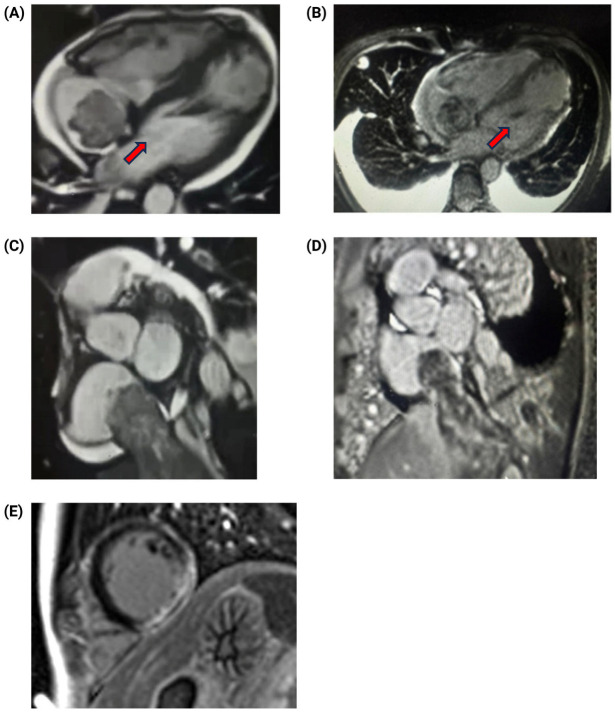
Cardiac magnetic resonance imaging. (**A**) T1-weighted horizontal four-chamber view showing a predominantly isointense signal throughout the mass, with irregular borders and heterogeneous appearance (arrow). (**B**) Four-chamber, late gadolinium-enhanced (LGE) T1-weighted PSIR view demonstrating moderate heterogeneous enhancement throughout the mass (arrow). (**C**) T1-weighted sagittal short-axis view revealing a mass with irregular borders and heterogeneous signal within the inferior vena cava. (**D**) Sagittal short-axis gadolinium-enhanced T1-weighted PSIR view showing moderate heterogeneous enhancement throughout the mass. (**E**) Subendocardial delayed enhancement indicative of myocardial infarction.

Three days post-surgery, the patient began chemotherapy with bleomycin, etoposide, and cisplatin (BEP regimen). However, due to a significant elevation in liver enzymes, with aspartate aminotransferase (AST) and alanine aminotransferase (ALT) levels reaching 944 U/L and 1507 U/L, respectively, the regimen was modified to cisplatin monotherapy, which was continued for five cycles. Subsequent transthoracic echocardiography (TTE) revealed a fixed, irregular mass in the right atrium. The mass did not extend into the right ventricle and did not appear to impair cardiac function. To further characterize the lesion, cardiac magnetic resonance (CMR) imaging was performed 7 days after surgery. CMR confirmed an irregular, heterogeneous mass in the right atrium and identified an apical infarction in the left ventricle, with non-viable myocardium in the lateral apical wall and segment 17, accounting for approximately 6% of myocardial fibrosis. Left ventricular ejection fraction (LVEF) was moderately reduced to 45%, and an apical aneurysm was noted in the right ventricle (Figure 3).

Considering these findings, heart failure management was initiated, including a combination of 5 milligrams of enalapril every 12 h, 6.25 milligrams of carvedilol every 12 h, 12.5 milligrams of spironolactone, and 10 milligrams of dapaglifozine every 24 h. The treatment was well tolerated, and the patient reported symptomatic improvement. In view of his thrombotic history and the presence of an intracardiac mass, rivaroxaban was added to his regimen for anticoagulation.

**Figure 4:**
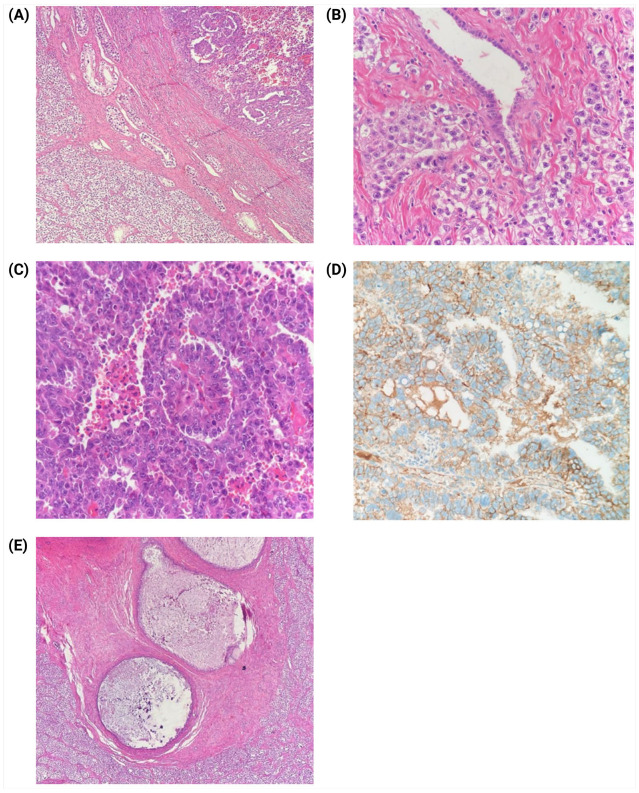
Histopathological analysis of testicular tumor. (**A**) Microscopic examination of the right testicle, revealing a mixed-germ cell tumor. On the left, a diffuse solid growth pattern of clear cells permeating seminiferous tubules, characteristic of the seminomatous component. On the right, a predominantly papillary growth pattern with areas of necrosis, indicative of embryonal carcinoma (H&E staining, 4×). (**B**) Seminoma infiltrating the rete testis, displaying a solid growth pattern composed of cells with well-defined membranes, clear cytoplasm, polygonal nuclei, and prominent nucleoli (H&E staining, 40×). (**C**) Embryonal carcinoma component characterized by papillary formations and solid areas, composed of polygonal cells with large, sometimes pleomorphic, nuclei, prominent nucleoli, frequent mitotic figures, and areas of necrosis with karyorrhexis (H&E staining, 40×). (**D**) Immunohistochemical staining for CD30 demonstrating positive expression in the cell membrane of the embryonal carcinoma component (CD30 staining, 40×). (**E**) Mature teratoma exhibiting mesenchymal tissue with cystic structures lined by gastrointestinal epithelium (H&E staining, 10×).

Histopathological analysis of the excised testicular tumor confirmed a mixed-germ cell tumor, composed of 75% seminoma, 20% embryonal carcinoma, and 5% post-pubertal teratoma (Figure 4).

**Figure 5:**
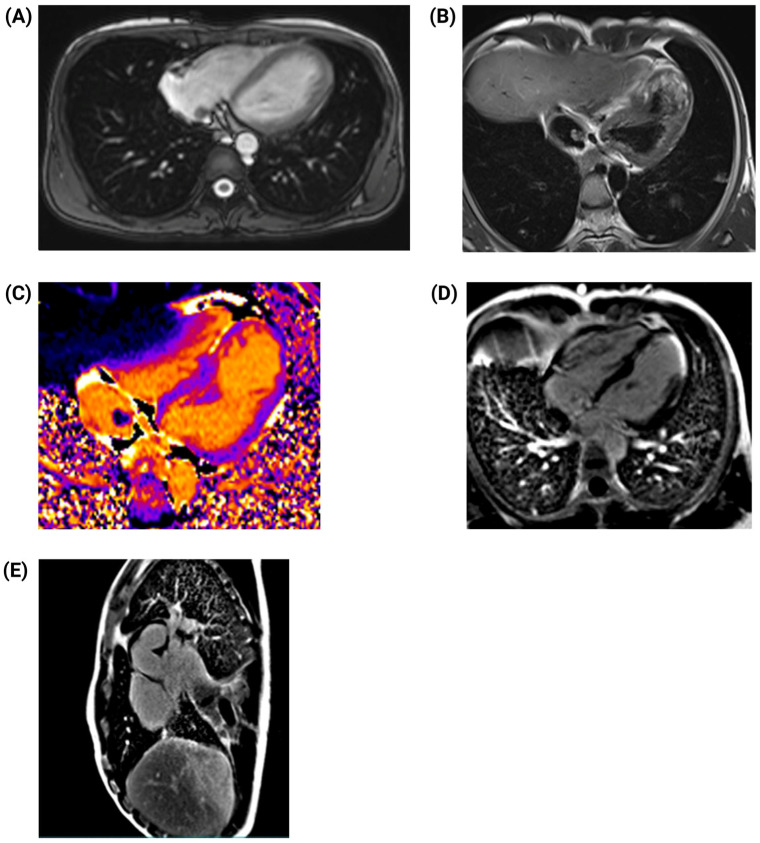
Follow-up cardiac magnetic resonance (CMR) imaging. (**A**) T1-weighted horizontal four-chamber view showing a reduction in the size of the intracardiac mass. (**B**) Double-inversion recovery pulse (black blood) T2-weighted imaging for anatomical evaluation and tissue characterization, demonstrating an isointense signal throughout the mass. (**C**) T1 mapping aiding in the differentiation between benign and malignant tumors. (**D**) Four-chamber, late gadolinium-enhanced (LGE) T1-weighted PSIR view, revealing a small residual mass attached to the wall of the right atrium and late transmural enhancement in the apical region (**E**) Sagittal short-axis gadolinium-enhanced T1-weighted PSIR view showing moderate heterogeneous enhancement throughout the mass.

Following three additional cycles of BEP-based chemotherapy, follow-up imaging showed a significant reduction in metastatic tumor burden. Both echocardiography and CMR confirmed a decrease in the size of the intracardiac mass. Tumor marker levels declined markedly, suggesting a favorable response to therapy. The most recent CMR scan identified a residual 5.5 × 3.5 cm^2^ lesion in the right atrium, which did not exhibit free-floating components or cause obstruction of the right ventricular outflow tract (Figure 5), indicating effective disease control.

At the two-month follow-up after initiating chemotherapy, transthoracic echocardiography demonstrated a marked improvement in left ventricular ejection fraction (LVEF), increasing from 45% to 60%. By the most recent clinical evaluation in March 2025, five months after diagnosis, the patient remained completely asymptomatic, with no signs of cardiovascular compromise or chemotherapy-related adverse effects. Serial measurements of serum tumor markers, including alpha-fetoprotein and beta-human chorionic gonadotropin, remained significantly decreased, indicating sustained therapeutic response (Table 1). Ongoing clinical and imaging surveillance is planned. Informed consent was obtained from the patient for publication of this report.

## 3. Discussion

**Figure 6:**
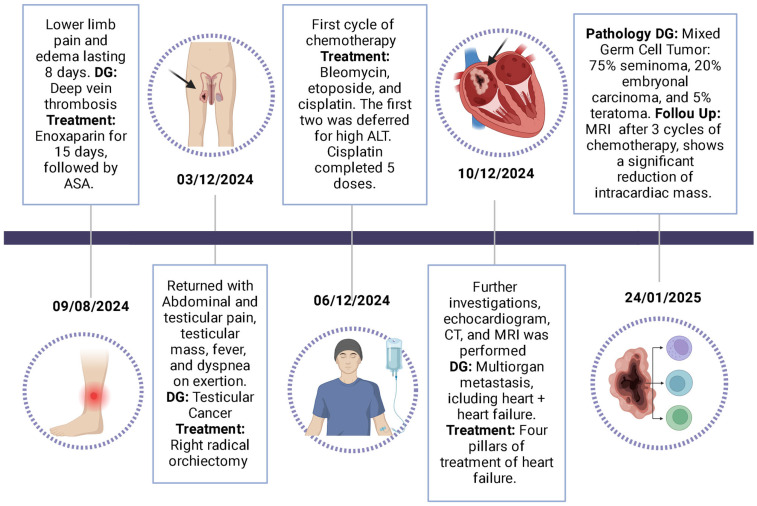
Patient timeline events.

We report the case of a young male with a testicular tumor and metastases to the lungs, liver, retroperitoneal lymph nodes, and an intracardiac mass extending into the right atrium. Histopathology confirmed a mixed-germ cell tumor (75% seminoma, 20% embryonal carcinoma, 5% teratoma). The patient underwent radical orchiectomy and BEP chemotherapy, resulting in a significant reduction in the intracardiac mass (Figure 6).

Testicular cancer accounts for 1–2% of all malignant tumors in men and is often associated with a hypercoagulable state, increasing the risk of thromboembolic events. Germ cell tumors (GCTs) constitute nearly 90% of all testicular malignancies, with seminomas being the most common subtype [6]. While testicular cancer frequently metastasizes to the lungs, liver, para-aortic and mediastinal lymph nodes, brain, and bones, intracardiac metastases remain exceedingly rare. Autopsy studies, such as the one by Bredael et al. [7], indicate that intracardiac metastases occur in less than 1% of patients with testicular carcinoma [3,6,8].

Primary cardiac tumors are rare, with an incidence of less than 0.02%, whereas secondary (metastatic) cardiac tumors are more common. However, intracavitary tumor growth remains an uncommon manifestation. The most frequent malignancies that metastasize the heart include lung and breast cancers, via mechanisms such as direct invasion, hematogenous dissemination, lymphatic spread, or intracavitary extension. In testicular cancer, hematogenous spread through the inferior vena cava is the primary mechanism for cardiac metastases [6,9].

Several risk factors predispose young patients to testicular cancer and its metastatic spread, including cryptorchidism, a family history of testicular cancer, and genetic syndromes such as Klinefelter syndrome. In this case, the patient’s history of deep vein thrombosis suggests a hypercoagulable state, potentially facilitating tumor spread and thrombus formation [6].

Intracavitary metastases can have severe clinical consequences, including heart failure, cardiogenic shock due to obstruction of blood flow, and cardioembolic events such as pulmonary embolism or stroke [5]. In some cases, these metastases are detected incidentally on imaging. Differential diagnoses include primary cardiac tumors (e.g., myxomas, sarcomas), thrombi, and other metastatic lesions. Advanced imaging modalities such as computed tomography (CT), magnetic resonance imaging (MRI), and positron emission tomography (PET-CT) play a crucial role in disease characterization and treatment planning. Histopathological analysis remains the diagnostic gold standard [8].

Metastatic tumors involving the endocardium and intracavitary structures often present as firm, white nodules adherent to the endocardial surface, most commonly in the right atrium or right ventricle. Alternatively, these metastases may manifest as loosely attached tumor emboli, leading to significant blood flow obstruction [3,10]. The histological morphology of the metastatic site usually mirrors that of the primary tumor.

Mixed-germ cell tumors originate from germ cell neoplasia in situ and contain more than one germ cell tumor component. The median age at presentation is 30 years, with embryonal carcinoma-predominant tumors occurring at an average age of 28 years, and seminoma-predominant tumors at 33 years [11]. Most patients present with a testicular mass, sometimes accompanied by pain. In cases with a substantial choriocarcinoma component, the likelihood of metastatic presentation is higher [12].

Histologically, the components of mixed-germ cell tumors resemble their pure counterparts. Embryonal carcinoma, teratoma, seminoma, and yolk sac tumor are the most frequent combinations. Choriocarcinoma-containing tumors often demonstrate a more aggressive clinical course and are diagnosed at a more advanced stage [13].

During tumorigenesis, malignant cells undergo metabolic reprogramming characterized by a shift toward increased glycolysis even under normoxic conditions, a process known as the Warburg effect. This metabolic adaptation leads to excessive lactate production, which fuels cellular proliferation and biosynthesis while contributing to an acidic tumor microenvironment that promotes invasion, immune evasion, and metastasis [14].

Key molecular mediators of this metabolic shift include monocarboxylate transporters (notably MCT4 and its chaperone CD147) and glucose transporters such as GLUT1 and GLUT3. Studies such as those by Silva et al. [15] have demonstrated that overexpression of MCTs correlates with enhanced tumor aggressiveness and poor prognosis in testicular germ cell tumors (TGCTs). Furthermore, Perri et al. [16] reported that embryonal carcinoma components within MGCTs exhibit higher expression of glycolysis-related markers compared to seminomatous elements, reinforcing the link between metabolic reprogramming and aggressive tumor phenotypes.

Unfortunately, in our case, immunohistochemical analysis of markers such as GLUT1, MCT1, MCT4, CD44, and other glycolysis-related proteins could not be performed due to the technical and financial limitations of the public healthcare system in Ecuador. Despite this, we acknowledge the prognostic and potential therapeutic value of these markers, particularly in aggressive forms of TGCTs, and we advocate for their inclusion in future clinical investigations and routine pathological assessments.

Notably, embryonal carcinoma exhibits extensive DNA methylation at non-canonical cytosine sites (e.g., CpA, CpT, CpC), correlating with tumor progression. Additionally, microRNAs have emerged as promising biomarkers for disease monitoring in germ cell tumors. Among these, miR-371a-3p is particularly relevant, as it is highly overexpressed in seminomas, embryonal carcinomas, and mixed-germ cell tumors, making it a potential serum biomarker for disease tracking and treatment response assessment [13,17].

Management of metastatic testicular GCTs requires a multimodal approach, including chemotherapy, surgery, and in selected cases, radiotherapy. In this case, three cycles of etoposide and cisplatin led to a significant reduction in the intracardiac tumor burden [6,9].

The 2025 National Comprehensive Cancer Network (NCCN) guidelines recommend platinum-based chemotherapy as the first-line treatment for metastatic GCTs [18]. Surgical intervention may be considered for residual or hemodynamically significant masses. Radiotherapy is primarily reserved for seminomas or palliative care. Surgical decisions for intracardiac metastases depend on the lesion’s size, anatomical location, and functional impact [6,19,20,21,22].

Testicular GCTs are among the most treatable malignancies, with five-year survival rates of approximately 90% for patients without distant metastases and 60% for those with metastatic disease. However, the presence of non-pulmonary visceral metastases, such as intracardiac involvement, is associated with a poorer prognosis, requiring intensive multidisciplinary follow-up [6,8,9,23].

Long-term follow-up is essential for detecting recurrence and managing late complications such as cardiomyopathy, secondary malignancies, or thromboembolic events. Regular imaging, tumor marker surveillance (e.g., alpha-fetoprotein and beta-human chorionic gonadotropin), and cardiac function assessments are essential components of follow-up care [6]. This case highlights the importance of recognizing atypical metastatic patterns in testicular cancer, emphasizing the role of advanced imaging, biomarker surveillance, and a multidisciplinary approach in achieving optimal patient outcomes.

## 4. Conclusions

This case illustrates a rare presentation of metastatic testicular GCT with intracardiac involvement, highlighting the importance of recognizing atypical metastatic patterns and their clinical implications. Despite the complexity of the disease, the patient demonstrated a favorable response to chemotherapy, reinforcing the efficacy of current treatment protocols for testicular GCTs. Long-term follow-up and a multidisciplinary approach remain crucial for monitoring recurrence and managing potential complications. This case adds to the expanding body of knowledge on rare metastatic manifestations and underscores the need for further research and clinical advancements in the diagnosis and management of testicular GCT metastases.

## Figures and Tables

**Table 1:** Clinical–hematological profile of the patient.

Test	Results	Reference Range
WBC	8.89 × 10^3^/μL	3.4–9.7 × 10^3^/μL
Neutrophil	6.53 × 10^3^/μL	2.2–4.8 × 10^3^/μL
Lymphocyte	1.75 × 10^3^/μL	1.1–3.2 × 10^3^/μL
Hemoglobin	14.9 g/dL	14.0–18.0 g/dL
Platelet	361,000/μL	130,000–400,000/μL
Lactate dehydrogenase (LDH)	1683 U/L	135–225 U/L
Human chorionic gonadotropin subunit b HCG-b	11.4 IU/L	<5 IU/L
a-fetoprotein (AFP)	3490 UI/mL	0–10 UI/mL
Post-surgery and first chemotherapy profile		
Lactate dehydrogenase (LDH)	261.00	135–225 U/L
a-fetoprotein (AFP)	429.00	0–10 UI/mL
Human chorionic gonadotropin subunit b HCG-b	0.2 IU/L	<5 IU/L

## Data Availability

Due to the nature of this case report, the data supporting the findings cannot be shared publicly in order to protect patient confidentiality. However, further details may be made available by the corresponding author upon reasonable request.
